# Premenopausal abnormal uterine bleeding and risk of endometrial cancer

**DOI:** 10.1111/1471-0528.14385

**Published:** 2016-10-20

**Authors:** ME Pennant, R Mehta, P Moody, G Hackett, A Prentice, SJ Sharp, R Lakshman

**Affiliations:** ^1^ Public Health DirectorateCambridgeshire County CouncilCambridgeUK; ^2^ Cambridge University Hospitals NHS Foundation trustCambridgeUK; ^3^ Department of Obstetrics & GynaecologyUniversity of CambridgeCambridgeUK; ^4^ Medical Research Council Epidemiology UnitUniversity of CambridgeCambridgeUK

**Keywords:** Biopsy, endometrial neoplasms, premenopause, risk, systematic review

## Abstract

**Background:**

Endometrial biopsies are undertaken in premenopausal women with abnormal uterine bleeding but the risk of endometrial cancer or atypical hyperplasia is unclear.

**Objectives:**

To conduct a systematic literature review to establish the risk of endometrial cancer and atypical hyperplasia in premenopausal women with abnormal uterine bleeding.

**Search strategy:**

Search of PubMed, Embase and the Cochrane Library from database inception to August 2015.

**Selection criteria:**

Studies reporting rates of endometrial cancer and/or atypical hyperplasia in women with premenopausal abnormal uterine bleeding.

**Data collection and analysis:**

Data were independently extracted by two reviewers and cross‐checked. For each outcome, the risk and a 95% CI were estimated using logistic regression with robust standard errors to account for clustering by study.

**Main results:**

Sixty‐five articles contributed to the analysis. Risk of endometrial cancer was 0.33% (95% CI 0.23–0.48%, *n* = 29 059; 97 cases) and risk of endometrial cancer or atypical hyperplasia was 1.31% (95% CI 0.96–1.80, *n* = 15 772; 207 cases). Risk of endometrial cancer was lower in women with heavy menstrual bleeding (HMB) (0.11%, 95% CI 0.04–0.32%, *n* = 8352; 9 cases) compared with inter‐menstrual bleeding (IMB) (0.52%, 95% CI 0.23–1.16%, *n* = 3109; 14 cases). Of five studies reporting the rate of atypical hyperplasia in women with HMB, none identified any cases.

**Conclusions:**

The risk of endometrial cancer or atypical hyperplasia in premenopausal women with abnormal uterine bleeding is low. Premenopausal women with abnormal uterine bleeding should first undergo conventional medical management. Where this fails, the presence of IMB and older age may be indicators for further investigation. Further research into the risks associated with age and the cumulative risk of co‐morbidities is needed.

**Tweetable abstract:**

Contrary to practice, premenopausal women with heavy periods or inter‐menstrual bleeding rarely require biopsy.

## Tweetable abstract

Contrary to practice, premenopausal women with heavy periods or inter‐menstrual bleeding rarely require biopsy.

## Introduction

Endometrial cancer has a world‐wide incidence of 9 per 100 000 women, with a 1% lifetime risk.^1^ Most cases are in women aged >50 years.^1^ Unopposed oestrogen exposure is a significant risk factor,^2^ where prolonged exposure causes continual endometrial proliferation and, potentially, endometrial carcinoma.^2^ Other factors influencing oestrogen exposure include obesity, polycystic ovarian syndrome (PCOS), anovulation, nulliparity, and type 2 diabetes mellitus^3^ and these are also thought to increase the risk of endometrial cancer. Endometrial hyperplasia, that is, irregular proliferation of the endometrial glands, may, in some cases, be a precursor to endometrial cancer. ‘Atypical’ hyperplasia poses the highest risk and, as with endometrial cancer, is managed with hysterectomy.^4^

Endometrial cancer most commonly presents with post‐menopausal bleeding (PMB).^3^ It is therefore recommended that women presenting with PMB are referred for further investigation.^5^, ^6^ Premenopausal abnormal uterine bleeding is common and estimated to interfere with daily life in more than one‐fifth of women.^7^ However, the current evidence base on premenopausal abnormal uterine bleeding and the risk of endometrial cancer is unclear.^8^ The selection of possible indicators for biopsy to exclude endometrial cancer is contentious^9^ and current guidance varies. All guidelines appear to recommend an age cutoff, above which patients are referred; in some it is 40^10^, ^11^ and in others it is 45 years.^12^, ^13^ All identify IMB as an indication for biopsy, and some also recommend biopsy based on other risk factors, such as obesity or PCOS.^13,11^ In some, such as the UK National Institute for Health and Care Excellence (NICE) guideline, it is recommended that biopsy should only be undertaken when conventional medical management has failed,^12^ whereas other guidance recommends direct referral for biopsy in higher risk (e.g. older) patients with abnormal uterine bleeding.^10^, ^11^, ^13^

In the UK, despite the NICE guideline, it is unclear whether all premenopausal women with abnormal uterine bleeding complete conventional management before referral for biopsy, or whether women considered ‘high risk’ are directly referred. Although guidelines, including the UK NICE guideline, were underpinned by some research evidence, none appears to have been based on a comprehensive literature review and there has been no other systematic review examining the risk of endometrial malignancy in this group. The aim of this work was therefore to conduct a systematic review to establish the risk of endometrial cancer and atypical hyperplasia in women with abnormal uterine bleeding and to make some judgment about the relative validity of current guidelines.

## Methods

### Search strategy

A search was performed in PubMed, Embase and the Cochrane library from database inception to August 2015 with terms related to abnormal uterine bleeding and terms for endometrial cancer or indications or methods for investigation (Appendix S1).

### Selection criteria

Prospective or retrospective studies of patients with abnormal uterine bleeding, where endometrial cancer or atypical hyperplasia was an outcome, were included in the review. Studies in populations of exclusively post‐menopausal women, and studies of mixed populations of pre and post‐menopausal women where the data could not be separated, were excluded. Study selection was undertaken by one reviewer.

### Data collection and quality assessment

Information on study and patient characteristics and numbers of individuals with endometrial cancer and atypical hyperplasia were extracted from included studies. Data was extracted independently by two reviewers, with any disagreements resolved by consensus or reference to a third reviewer. Quality assessment was conducted by one reviewer, examining the internal and external validity of risk estimates. Internal validity was judged by the accuracy of the method used to investigate for the presence of malignancy. Where histological testing was conducted for all women, internal validity was judged to be good. In studies where a pathological diagnosis was not present for all women, internal validity was also potentially good where investigators appeared to have implemented adequate referral/treatment pathways. External validity was assessed as the applicability of the study sample to the population of this review i.e. women presenting in primary care with abnormal uterine bleeding. Where studies included women who appeared to have been included in the study because of high suspected risk, these were judged as less applicable, with low external validity.

### Data analysis

The risk was calculated using logistic regression with robust standard errors to account for clustering by study. Analyses were conducted for rates of (i) endometrial cancer and (ii) endometrial cancer and atypical hyperplasia combined. Sensitivity analysis was conducted, including only those studies judged to have good internal and external validity. The risk of endometrial cancer was also estimated within subgroups of studies defined by location (Western/non‐Western), study design (prospective/retrospective), and size (≥100 versus <100 premenopausal women). Where studies reported data separately for premenopausal women of different ages, these were included in an additional analysis estimating the risk of endometrial cancer in women aged <40, 40–50 and >50 years.

## Results

### Study characteristics

**Figure 1:**
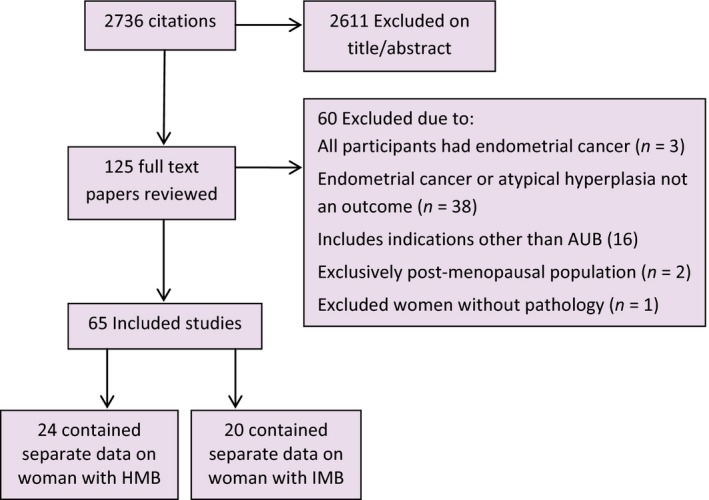
PRISMA flow diagram.

Of 2736 original articles retrieved, 125 were obtained as full papers; 60 were excluded as indicated in Figure 1. Sixty‐five studies (*n* = 29 059 premenopausal women with abnormal uterine bleeding) were included and their characteristics and study risk of endometrial cancer are shown in Table S1. Studies were conducted in Europe (*n* = 32), North America (*n* = 6), Australasia (*n* = 2) and non‐Western countries (*n* = 25). Most were in populations of women undergoing investigation exclusively for abnormal bleeding, but some included women undergoing investigation for other indications (data for women with bleeding problems was extracted separately). The mean age in the majority of studies was between 40 and 50 years.

### Study quality

The internal validity of studies was generally considered to be good. In most, the majority of women had undergone histopathological testing and it appeared likely that the majority of cases of malignancy were identified. Fifty‐three studies were judged to have good internal validity, but 12 were considered to be potentially less reliable. The external validity of studies was generally considered to be lower. Many were retrospective (*n* = 37), including women who had been investigated with invasive testing such as endometrial biopsy or dilation and curettage. The reason for further investigation in these populations was not often reported but where it was, the reasons were suspected risk factors such as obesity, anovulation or previous oligomenorrhoea,^14^ older age or failed medical management.^15^, ^16^, ^17^ However, some studies were judged to be more externally valid. Forty prospective studies, and retrospective studies where not all the population had undergone invasive testing, were judged to be potentially more applicable as the population may have been more similar to women presenting in primary care. Twenty‐five studies were judged as having poor external validity.

### Risk of endometrial cancer

Risk of endometrial cancer according to menstrual status, based on aggregated numbers of events and individuals across studies, is shown in Table 1. In all studies of premenopausal women with abnormal uterine bleeding (*n* = 65), the risk was low, at 0.33% (95% CI 0.23–0.48%, *n* = 29 059; 97 cases). When data for subpopulations of premenopausal women were separated, the risk of endometrial cancer was lower for women with HMB 0.11% (95% CI 0.04–0.32%, *n* = 8352; 9 cases; 24 studies) than for women with IMB 0.52% (95% CI 0.23–1.16%, *n* = 3109; 14 cases; 20 studies).

**Table 1:** Prevalence of endometrial cancer and atypical hyperplasia in populations of premenopausal women with abnormal uterine bleeding

	Number of studies	Number of cases	*n*	%	Lower 95% CI	Upper 95% CI
**Endometrial cancer**						
AUB	65	97	29 059	0.33	0.23	0.48
HMB	24	9	8352	0.11	0.04	0.32
IMB	20	14	3109	0.52	0.23	1.16
AUB sensitivity analysis^a^	29	40	14 511	0.28	0.15	0.50
**Endometrial cancer or atypical hyperplasia** b						
AUB^c^	31	207	15 772	1.31	0.96	1.80

AUB, abnormal uterine bleeding; HMB, heavy menstrual bleeding; IMB, inter‐menstrual bleeding.

a Includes only studies judged to have higher internal and external validity.

b Includes only studies reporting rates of both endometrial cancer and atypical hyperplasia.

c In five studies that reported rates of atypical hyperplasia separately for women with HMB, none reported any cases. No studies reported rates of atypical hyperplasia separately for women with IMB.

### Risk of endometrial cancer or atypical hyperplasia

Rates for the risk of endometrial cancer or atypical hyperplasia are also shown in Table 1. Thirty‐one studies reported rates of atypical hyperplasia and endometrial cancer/atypical hyperplasia showed a combined risk of 1.31% (95% CI 0.96–1.80, *n* = 15 772, 207 cases). Only five studies reported rates of atypical hyperplasia separately for women with HMB and all reported that there were no cases of atypical hyperplasia. No studies reported rates of atypical hyperplasia separately for women with IMB.

### Risk by age group

**Figure 2:**
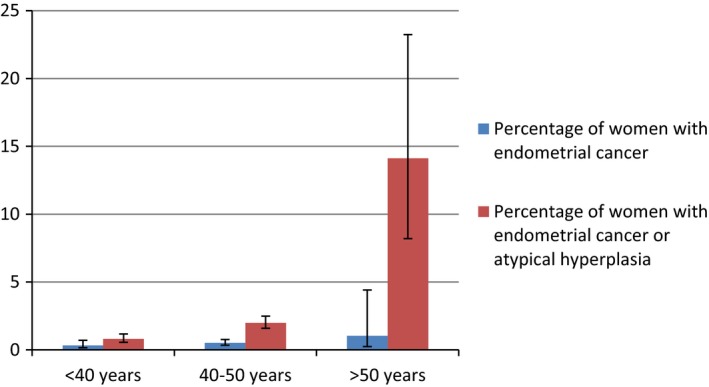
Prevalence of endometrial cancer and atypical hyperplasia in pre‐menopausal women with abnormal uterine bleeding in different age groups.

Twelve studies reported rates of endometrial cancer and four studies reported rates of both endometrial cancer and atypical hyperplasia in specific age groups (Figure 2). For endometrial cancer alone, there was an increase with age group but confidence intervals were wide and there were no conclusive differences between groups: <40 years 0.33% (95% CI 0.16–0.70%, *n* = 2401; 8 cases), 40–50 years 0.51% (95% CI 0.34–0.77%, *n* = 6662; 31 cases) and >50 years 1.04% (95% CI 0.24–4.41, *n* = 277, 2 cases). However, for the risk of endometrial cancer or atypical hyperplasia, the increase with age group was significant: <40 years 0.81 (95% CI 0.56–1.17, *n* = 1240; 10 cases), 40–50 years 1.99% (95% CI 1.59–2.48, *n* = 5131, 102 cases) and >50 years 14.12% (95% CI 8.20–23.24, *n* = 85; 12 cases).

### Sensitivity and subgroup analysis

In the sensitivity analysis (Table 1), including only studies that were judged to have higher internal and external validity (*n* = 29) the estimated risk of endometrial cancer in all premenopausal women was 0.28% (95% CI 0.15–0.50, *n* = 14 511; 40 cases), suggesting that the findings from the main analysis were robust. In the subgroup analysis, estimates of risk were similar in subgroups defined by study size (<100 versus ≥100), design (prospective versus retrospective) or location (Westernised versus non‐Westernised) (Figure S1).

## Discussion

### Main findings

This review aimed to estimate the risk of endometrial cancer and atypical hyperplasia in premenopausal women presenting with abnormal uterine bleeding and to make some judgement about whether current guidelines are appropriate. Given the large numbers of women presenting in primary care, and the potential for complications, anxiety and wasted healthcare resources in undertaking unnecessary biopsies, estimating this risk is important. NICE use a > 3% cancer risk to underpin screening guidance.^6^ Where risk is anticipated to be higher, guidance recommends that patients be referred for further testing. The overall estimated risk of endometrial cancer or atypical hyperplasia in premenopausal women in this review was only 1.33%, with confidence intervals not reaching the NICE recommended 3% risk (95% CI 0.96–1.80).

The risk of endometrial cancer in women with HMB was particularly low (0.11%, 95% CI 0.04–0.32) and no cases of atypical hyperplasia were observed in studies of women with HMB. The risk of endometrial cancer was slightly higher in women with IMB (0.52%, 95% CI 0.23–1.16) and IMB may represent a symptom of endometrial cancer. The number of cases of atypical hyperplasia was not reported separately for women with IMB and it was therefore not possible to assess the overall endometrial cancer/atypical hyperplasia risk. However, if it is assumed that risk in women with HMB is lower than that of the whole premenopausal group, it may be assumed that the risk in women with IMB is likely to be greater than the whole premenopausal group, i.e. women with IMB will have a greater than 1.33% risk of endometrial cancer/atypical hyperplasia. Therefore, for women with persistent IMB who are not responding to medical management, referral for further testing may be appropriate.

There were some data regarding the association of age with risk of endometrial cancer and atypical hyperplasia. There was an increase in risk for women aged 40–50 years compared with <40 years that was significant when considering endometrial cancer and atypical hyperplasia together. Although the numbers of studies contributing to these analyses was very small (only four for endometrial cancer and atypical hyperplasia) it appears likely that there is some increase in risk associated with increased age during the 40s. The NICE guideline recommends age as a criterion for biopsy only after all conventional medical management has failed.^12^ Given that the upper confidence interval of estimated risk in the 40‐ to 50‐year age group was below the 3% risk cutoff, the NICE guidance appears justified. Due to the current lack of data, it is not possible to identify a specific age at which there may be an increase in risk, but further research may help to determine an appropriate age criteria for referral following failure of conventional medical management.

The current review did not find data on risks associated with co‐morbidities but we reviewed the literature. Mutations related to HNPCC, or Lynch syndrome, are associated with a high lifetime risk of endometrial cancer (12–44%);^18^ this is an important consideration, but only in this specific and small group of women.

For other risk factors, the association appears less pronounced. A recent meta‐analysis showed PCOS to be a significant risk factor for endometrial cancer (odds ratio 2.79),^19^ but all included studies were case‐control studies and hence this estimate is likely to be unreliable. There is more robust evidence for other risk factors from systematic reviews of prospective cohort studies. Higher BMI was associated with increased risk of endometrial cancer, with a relative risk (RR) of 1.54 for a five‐point increase in BMI and RR 1.41 in the subgroup of studies of premenopausal women.^20^ For women with diabetes mellitus, risk of endometrial cancer was higher compared with those without diabetes mellitus (RR 1.89),^21^ and being parous as opposed to nulliparous was estimated to decrease the risk of endometrial cancer (RR 0.69).^22^

Caution is needed in relating these findings as studies are of general populations of women, and not specifically in women with bleeding problems. It is therefore unclear whether the relative risks would be the same. It is also unclear to what extent risks factors are independent or whether they confound one another. It appears unlikely that the risks are cumulative and treating them as such is likely to overestimate total relative risk.

However, even without these cautions, given the low risk of endometrial cancer, the absolute risks are likely to be low. For example, considering BMI, for a woman presenting with HMB, her risk of endometrial cancer increases from 0.11% (assuming risk found in the current review and a 1.41 increase in risk^20^) to 0.16% with a five‐point increase in BMI and to 0.22% with a ten‐point increase.

### Strengths and limitations

This review included a large number of studies and provides a robust estimate of the risk of endometrial cancer in premenopausal women with abnormal uterine bleeding. No other systematic review was found and this may be the first to undertake this type of work. It is clinically meaningful and has direct implications for practice.

A limitation of this review is that studies predominately included populations of women referred to secondary care who were undergoing invasive testing (dilation and curettage or endometrial biopsy) for suspicion of abnormalities or pathology. They are therefore unlikely to reflect typical populations of women presenting in primary care. Women undergoing invasive testing for suspected pathology may have a higher incidence compared with general populations of women presenting with abnormal uterine bleeding and these studies may thus overestimate the risk of endometrial cancer. The findings of the review are therefore likely to be conservative, further supporting the view that premenopausal abnormal uterine bleeding confers a low risk for endometrial cancer or atypical hyperplasia.

Another limitation of the review was the difficulty in searching for studies. Most studies did not set out to determine rates of endometrial cancer as a primary outcome and results are incidentally reported. It is therefore possible that some studies were missed. However, a search strategy was used where endometrial cancer was not specified as a required outcome, and it appears likely that the majority of studies with relevant data were obtained.

### Interpretation

There generally appears to be a low risk of endometrial cancer in premenopausal women and much current guidance appears over‐cautious. The majority of women in studies were aged >40 years and overall risk of endometrial cancer was only 0.33%. When cases of atypical hyperplasia were also considered, the rate was still well below the NICE 3% threshold for cancer referrals.

HMB conferred a particularly low risk and although studies suggest that some co‐morbidities increase the risk of endometrial cancer, given the very low baseline risk associated with HMB, the absolute risk in the presence of co‐morbidities is likely to still be low. Guidance recommending direct referral for biopsy in premenopausal women >40 years^10^, ^11^ or with co‐morbidities^11^, ^13^ therefore appears unwarranted.

The risk associated with IMB appears to be slightly higher. It is unclear whether the cumulative risk of endometrial cancer and atypical hyperplasia would reach the 3% risk NICE recommend for cancer referral but, where medical management fails, referral of women with IMB may be justified.

The UK NICE guidance^12^ is the least cautious guideline and appears to provide a reasonable model, where women are only considered for biopsy in the presence of persistent IMB or, for women aged >45 years with HMB alone, following failure of conventional medical management. There is currently no evidence to suggest that clinicians should deviate from this.

## Conclusion

This review demonstrates that the risk of endometrial cancer or atypical hyperplasia in premenopausal women with abnormal uterine bleeding is low. Consequently, this group of women should first undergo conventional medical management. In women who have failed medical management, the presence of persistent IMB and older age may be indicators for further investigation. The review suggests that practice needs to change, where referral for biopsy in premenopausal women is only in selected cases. Further research is needed to better understand the effect of age and the cumulative effect of co‐morbidities.

### Disclosure of interests

None declared. Completed disclosure of interests form available to view online as supporting information.

### Contribution to authorship

MP and RL conceived the study. MP, PM and RM collected the data. SS analysed the data. MP, RL, GH and AP interpreted the data. MP, RL and RM drafted the manuscript. All authors commented on drafts of the manuscript and approved the final version.

### Funding

The work was undertaken under the auspices of the Centre for Diet and Activity Research (CEDAR), a UKCRC Public Health Research Centre of Excellence which is funded by the British Heart Foundation, Cancer Research UK, Economic and Social Research Council, Medical Research Council, the National Institute for Health Research, and the Wellcome Trust.

## Supporting information


**Figure S1.** Subgroup analysis.

**Table S1.** Study characteristics and prevalence of endometrial cancer in premenopausal women.

**Appendix S1.** Search strategies.
