# Supplementary figures and images for: Premenopausal abnormal uterine bleeding and risk of endometrial cancer
Source: BJOG. 2016 Oct 20;124(3):404–11. doi: 10.1111/1471-0528.14385 (PMC5297977; doi:10.1111/1471-0528.14385)

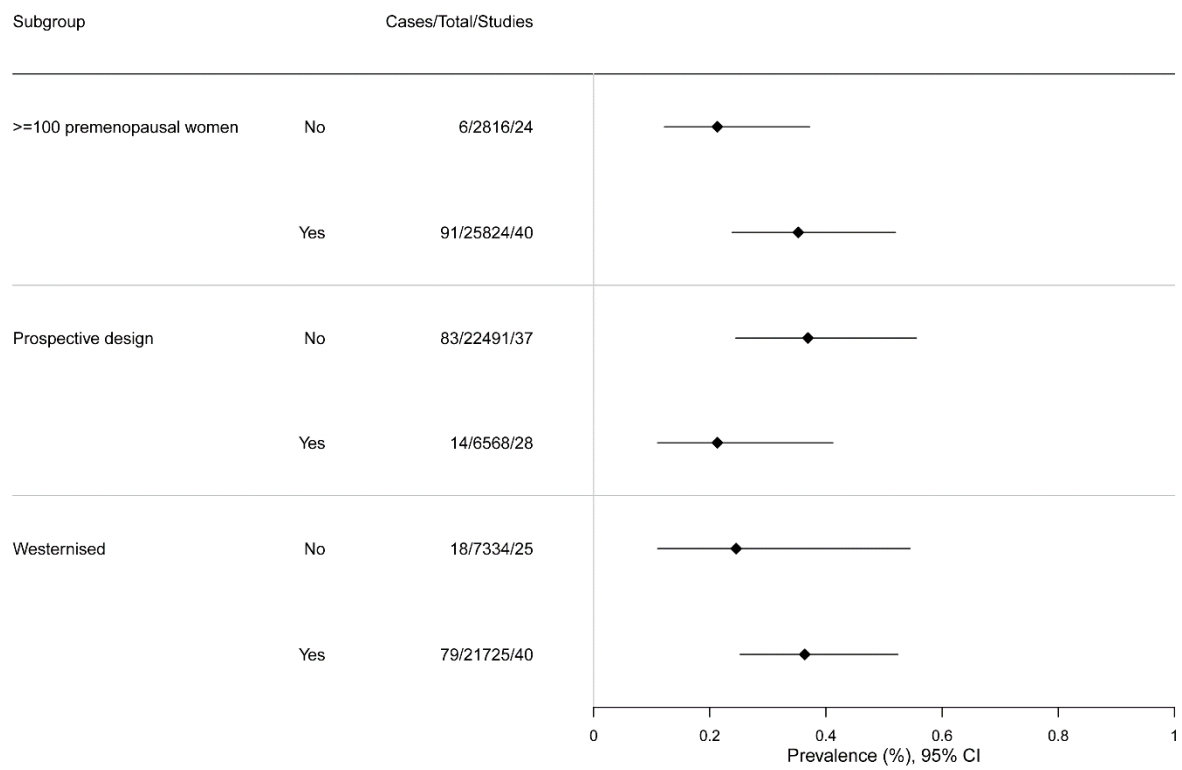

**Figure S1.** Subgroup Analysis

Supplement: Supplementary file 1 — Figure S1. Subgroup analysis. [file BJO-124-404-s001.pdf]
