# Supplementary material for: Premenopausal abnormal uterine bleeding and risk of endometrial cancer
Source: BJOG. 2016 Oct 20;124(3):404–11. doi: 10.1111/1471-0528.14385 (PMC5297977; doi:10.1111/1471-0528.14385)
Supplement: Supplementary file 2 — Table S1. Study characteristics and prevalence of endometrial cancer in premenopausal women. [file BJO-124-404-s002.pdf]

Table S1. Study characteristics and prevalence of endometrial cancer in premenopausal women

	Country	Design	Popuulation	Outcome measure	Premenopausal women	
					EC (%)	N
Akkad 1995[23]	UK	Retrospective	Pre and post menopausal women investigated for AUB	Diagnostic hysteroscopy	0 (0)	92
Alexopoulos 1999[24]	UK	Retrospective	Pre and post menopausal women investigated for AUB	Hysteroscopy, sometimes with biopsy	3 (0.17)	1,719
Allen 1990[25]	Tasmania	Retrospective	Pre and post menopausal women investigated for AUB	D&C, sometimes with hysteroscopy	4 (0.11)	3,780
Ash 1996[26]	Canada	Retrospective	Premenopausal women investigated for AUB	Endometrial biopsy	0 (0)	310
Bain 2002[27]	UK	Prospective	Premenopausal women investigated for AUB	Endometrial biopsy with/ without hysteroscopy	0 (0)	349
Bronz 1997[28]	Switzerland	Prospective	Premenopausal women investigated for AUB	TVS, SCHS and diagnostic hysteroscopy	0 (0)	83
Chawla 2014[29]	India	Prospective	Pre and post menopausal women investigated for AUB	TVS, SIS and hysteroscopy with biopsy	0 (0)	29
Clevenger-Hoeft 1999[30]	USA	Retrospective	Premenopausal women investigated for AUB	Sonohysterography, where indicated, biopsy	0 (0)	80
Crtichley 2004[31]	UK	Prospective	Pre and post menopausal women investigated for AUB	Endometrial biopsy	3 (0.62)	483
Damle 2013[32]	India	Retrospective	Pre and post menopausal women investigated for AUB	endometrial sampling	0 (0)	80
Dangal 2003[33]	Nepal	Retrospective	Pre and post menopausal women investigated for AUB	D&C and endometrial biopsy	0 (0)	39
De Angelis 2013[34]	Italy	Prospective	Premenopausal women investigated for HMB	Office hysteroscopy	1 (0.85)	118
De Kroon 2004[35]	The Netherlands	Prospective	Pre and post menopausal women investigated for AUB	Sugical hysteroscopy	0 (0)	41
de Vries 2000[36]	The Netherlands	Prospective	Premenopausal women investigated for AUB	Hysteroscopy and endometrial biopsy	0 (0)	62

Decloedt 1999[37]	UK	Retrospective	Pre and post menopausal women investigated for AUB	Hysteroscopy with biopsy where indicated	2 (0.43)	469
Dreisler 2009[38]	Denmark	Prospective	Women from the Danish Civil Registration System	TVS, SCSH, hysteroscopic resection where indicated	1 (0.50)	199
Dueholm 2001[14]	Denmark	Prospective	Premenopausal women investigated for AUB	SCSH, TVS, operative hysteroscopy where indicated	0 (0)	452
Dunn 2001[39]	USA	Prospective	Pre and post menopausal women investigated for AUB	Risk-stratified pathway, including endometrial biopsy	0 (0)	570
Emanuel 1995[40]	The Netherlands	Retrospective	Pre and post menopausal women investigated for AUB	Diagnostic hysteroscopy	4 (0.44)	908
Emmanuel 1995b[41]	The Netherlands	Retrospective	Pre and post menopausal women investigated for AUB	TVS and diagnostic hysteroscopy	0 (0)	213
Erdem 2007[42]	Turkey	Prospective	Pre and post menopausal women investigated for AUB	TVS and SIS	0 (0)	95
Esmer 2014[43]	Turkey	Retrospective	Premenopausal women investigated for AUB	Endometrial biopsy	16 (0.64)	2,516
Fakhar 2008[44]	Pakistan	Prospective	Pre and post menopausal women investigated for AUB	Pipelle biopsy followed by D&C	0 (0)	89
Farquhar 1999[45]	New Zealand	Retrospective	Premenopausal women with AUB undergoing	Endometrial biopsy	5 (0.48)	1,033
Fraser 1992[46]	Australia	Retrospective	Premenopausal women investigated for HMB	Some had hysteroscopy	0 (0)	316
Getpook 2006[47]	Thailand	Prospective	Premenopausal women investigated for IMB	Uterine curettage	4 (3.6)	111
Goldstein 1997[48]	USA	Retrospective	Premenopausal women investigated for AUB	Ultrasound. Biopsy or D&C in some cases	0 (0)	433
Gorleo 2008[49]	Italy	Prospective	Premenopausal women investigated for AUB	TVS and SCHS	0 (0)	61
Goyal 2015[50]	India	Prospective	Premenopausal women investigated for AUB	TVS and diagnostic hysteroscopy	0 (0)	100
Gulumser 2010[51]	UK	Retrospective	Pre and post menopausal women investigated for AUB	Hysteroscopy, biopsy in most cases	4 (0.90)	446
Hammouda	Saudi Arabia	Retrospective	Pre and Peri menopausal	Curettage, biopsy or	4 (0.61)	660

1967[52]			women investigated for AUB	hysterectomy		
Hauge 2010[53]	Norway	Prospective	Pre-menopausal women investigated for AUB refractory to medical treatment	TVS, SIS and hysteroscopy with biopsy	0 (0)	104
Hunter 2001[54]	UK	Retrospective	Pre and post menopausal women investigated for AUB	Hysteroscopy and biopsy	0 (0)	57
Iram 2010[9]	UK	Retrospective	Premenopausal women investigated for AUB	Endometrial biopsy	20 (0.67)	3,006
Jetley 2013[55]	India	Retrospective	Perimenopausal women investigated for AUB	Endometrial biopsy	0 (0)	219
Litta 1996[56]	Italy	Retrospective	Pre and post menopausal women investigated for persistent AUB	Hysteroscopy and biopsy	6 (1.59)	378
Loffer 1989[57]	USA	Retrospective	Pre and post menopausal women investigated for AUB	Endometrial biopsy	0 (0)	140
MacKenzie 1978[58]	UK	Retrospective	Pre and post menopausal women investigated for AUB	D&C	0 (0)	580
Machado 2005[59]	Oman	Prospective	Pre and post menopausal women investigated for AUB	TVS, hysteroscopy and endometrial biopsy	0 (0)	134
Masood 2015[60]	Pakistan	Prospective	Pre and post menopausal women investigated for AUB	Endometrial biopsy	1 (1.92)	52
Matthew 2010[61]	Oman	Retrospective	Pre and post menopausal women investigated for AUB	TVS, SISH and diagnostic hysteroscopy	0 (0)	54
Mortakis 1997[62]	Greece	Prospective	Pre and post menopausal women investigated for AUB	TVS with aspiration pipelle biopsy	1 (0.82)	122
Motashaw 1990[16]	India	Retrospective	Pre and post menopausal women investigated for AUB	Diagnostic hysteroscopy	1 (0.30)	328
Mukhopadhayay 2007[63]	India	Retrospective	Perimenopausal women investigated for AUB	TVS and hysteroscopy with biopsy	0 (0)	85
Nagele 1996[17]	UK	Retrospective	Pre and post menopausal women investigated for AUB	Diagnostic hysteroscopy	4 (0.21)	1,925
Ossola 1999[64]	Italy	Retrospective	Pre and post menopausal	TVS and diagnostic	0 (0)	33

			women investigated for AUB	hysteroscopy		
Paschopoulos 2001[65]	Greece	Retrospective	Pre and post menopausal women investigated for AUB	According to protocol (including TVS, hysteroscopy and biopsy)	2 (0.63)	316
Refaie 2005[66]	UK	Retrospective	Pre and post menopausal women investigated for AUB	Outpatient hysteroscopy and pipelle biopsy	0 (0)	68
Sadaf 2014[15]	Pakistan	Prospective	Pre and post menopausal women investigated for AUB after failed medical management	D&C	0 (0)	79
Shaheen 2005[67]	Pakistan	Prospective	Premenopausal women investigated for HMB	D&C	1 (0.83)	121
Soleymani 2014[68]	Iran	Retrospective	Pre and post menopausal women investigated for AUB	D&C	2 (0.42)	480
Stamatellos 2005[69]	Greece	Retrospective	Perimenopausal women using of HRT investigated for AUB	Endometrial biopsy	0 (0)	76
Subhankar 2011[70]	India	Prospective	Premenopausal women investigated for AUB	Hysteroscopy and endometrial biopsy	0 (0)	252
Svirsky 2008[71]	Israel	Retrospective	Premenopausal women investigated for AUB	Hysteroscopy and endometrial biopsy	0 (0)	218
Tehranian 2015[72]	Iran	Prospective	Premenopausal women investigated for AUB	SCHS, hysteroscopy and endometrial biopsy	0 (0)	90
Torrejon 1997[73]	Spain	Retrospective	Pre and post menopausal women investigated for AUB	Diagnostic hysteroscopy and D&C	4 (0.50)	802
Vale 1981[74]	USA	Retrospective	Pre and post menopausal women investigated for AUB	Hysteroscopy and D&C	0 (0)	419
Van den Bosch 2015[75]	UK	Prospective	Pre and post menopausal women investigated for AUB	TVS and SIS	1 (0.13)	763
Van Trotseburg 2000[76]	Austria	Retrospective	Premenopausal women investigated for AUB	Hysteroscopy, biopsy where indicated	0 (0)	317
Vercellini 1997[77]	Italy	Retrospective	Premenopausal women investigated for HMB	TVS and hysteroscopy with endometrial biopsy	2 (0.26)	770
Vercellini 1998[78]	Italy	Retrospective	Premenopausal women	TVS and biopsy	0 (0)	102

			investigated for HMB			
Yavuz 2012[79]	Turkey	Retrospective	Pre and post menopausal women investigated for AUB	TVS, endometrial sampling and curettage	0 (0)	52
Yildizhan 2008[80]	Turkey	Prospective	Pre and post menopausal women investigated for AUB	TVS, SIS and D&C, hysteroscopy or hysterectomy	0 (0)	79
Yumru 2009[81]	Turkey	Prospective	Pre and post menopausal women investigated for AUB	TVS, D&C and hysteroscopy	0 (0)	75
Zaman 2013[82]	Pakistan	Prospective	Pre and post menopausal women investigated for AUB	Pipelle biopsy	1 (1.92)	52

*Or other measure if mean was not available. For studies only reporting the age of mixed populations of pre and post-menopausal women, where the age of the pre-menopausal subgroup was not available NR (not reported) is stated.

AUB=abnormal uterine bleeding; D&C=dilation and curettage; HMB=heavy menstrual bleeding; IMB=intermenstrual bleeding; MP=menopausal; TVS=Transvaginal sonography; SCHS=saline contrast hysterosonography; SIS=Saline infusion sonography
