# Supplementary material for: Premenopausal abnormal uterine bleeding and risk of endometrial cancer
Source: BJOG. 2016 Oct 20;124(3):404–11. doi: 10.1111/1471-0528.14385 (PMC5297977; doi:10.1111/1471-0528.14385)
Supplement: Supplementary file 3 — Appendix S1. Search strategies. [file BJO-124-404-s003.pdf]

Appendix S1. Search strategies

PubMed

((((((((((((((((((((endometrial cancer[Title/Abstract]) OR ((cancer[Title/Abstract]) AND ((((uterus[Title/Abstract]) OR womb[Title/Abstract]) OR uterine[Title/Abstract]) OR endometrium[Title/Abstract])))))) OR sonohysterography[Title/Abstract]) OR (Dilatation[Title/Abstract] AND curettage[Title/Abstract])) OR ultrasound[Title/Abstract]) OR hysterectomy[Title/Abstract]) OR endometrial ablation[Title/Abstract]) OR endometrial resection[Title/Abstract]) OR laparoscopy[Title/Abstract]) OR Hysteroscopies[Title/Abstract]) OR biopsy[Title/Abstract]) OR sonography[Title/Abstract]) OR Pipelle[Title/Abstract]) OR Ultrasonography[Title/Abstract]) OR hysteroscopy[Title/Abstract]) OR pathology[Title/Abstract])) AND ((((((((heavy menstrual bleeding[Title/Abstract]) OR menorrhagia[Title/Abstract]) OR heavy periods[Title/Abstract]) OR abnormal uterine bleeding[Title/Abstract]) OR atypical uterine bleeding[Title/Abstract]) OR dysfunctional uterine bleeding[Title/Abstract]) OR Menometrorrhagia[Title/Abstract]))) Filters: Humans

Conducted 20/08/2015: 2,521 citations

Embase

("endometrial cancer" OR "dilatation and curettage" OR biopsy OR "endometrial resection" OR Hysteroscopies OR Pipelle OR hysteroscopy).ti,ab AND ("heavy menstrual bleeding" OR menorrhagia OR "heavy periods" OR "abnormal uterine bleeding" OR "atypical uterine bleeding" OR "dysfunctional uterine bleeding" OR Menometrorrhagia).ti,ab [Limit to: Human]

Conducted 19/10/2015: 1,700 citations

After duplicates removed = 2,736 citations
